# Medication- and Non-Medication-Related Causes of Relapse in First-Episode Psychosis Patients Admitted to Sultan Qaboos University Hospital

**DOI:** 10.7759/cureus.81125

**Published:** 2025-03-24

**Authors:** Yamamh M Al-Jubori, Al Zahra Al-Maskari, Hassan Mirza, Amira Al-Hosuni

**Affiliations:** 1 Psychiatry and Behavioral Sciences, Sultan Qaboos University, Muscat, OMN; 2 Behavioral Medicine, Sultan Qaboos University Hospital, Muscat, OMN

**Keywords:** drug compliance, first-episode psychosis, oman, psychosocial factors, relapse rate

## Abstract

Background

First-episode psychosis (FEP) patients often respond well to treatment; however, they remain at high risk of relapse. The factors contributing to relapse have been understudied, particularly in the Middle East.

Aim

This study aimed to identify both medication- and non-medication-related factors associated with relapse in FEP patients admitted to the Department of Behavioral Medicine (inpatient or outpatient) at Sultan Qaboos University Hospital (SQUH).

Methods

This retrospective cohort study included 213 Omani FEP patients aged 12-55 years who were treated at the inpatient or outpatient clinic of the Department of Behavioral Medicine at SQUH. Medical records from June 2006 to December 2019 were reviewed to assess relapse rates and associated sociodemographic and clinical factors.

Results

The average relapse rate among FEP patients was 14.28 months. Place of residence and left against medical advice (LAMA) were significantly associated with relapse, whereas factors such as seeking traditional healing, follow-up patterns, substance abuse, life events, and hospitalization status did not show a significant impact. Medication noncompliance was prevalent among nearly half of the patients, with an average relapse rate of 12 months - significantly shorter than the 17.22-month relapse rate observed in compliant patients. Regular follow-up was strongly linked to medication adherence, with patients who maintained consistent follow-ups demonstrating significantly higher compliance rates.

Conclusions

This study highlights key factors influencing relapse in FEP patients in Oman. Place of residence and LAMA were significantly associated with increased relapse risk. Medication adherence played a critical role, with noncompliance notably accelerating relapse. Regular follow-ups emerged as a crucial factor in maintaining medication adherence. Our findings emphasize the need for targeted interventions that consider the unique sociodemographic and clinical characteristics of FEP patients in Oman. The study cohort included 213 patients, predominantly female (57.3%, n = 122) and single (65.7%), with a mean age of 25 years (range: 12-55 years). Addressing these demographic factors can help develop strategies to reduce relapse rates and improve long-term outcomes.

## Introduction

Psychosis is a common and disruptive symptom associated with various psychiatric, neurological, and medical conditions. As a result, it has become a key focus in neuropsychiatric evaluation and treatment [1]. The term psychosis describes a state in which an individual loses touch with reality, experiencing disturbed thoughts and perceptions that make it difficult to differentiate between real and unreal experiences. Hallucinations and delusions are hallmark symptoms, often accompanied by incoherent speech and disrupted sleep patterns [2].

First-episode psychosis (FEP) refers to the first occurrence of a psychotic episode in an individual. During FEP, patients experience profound detachment from reality, leading to distress and disorientation. Cognitive function during this period varies depending on the severity of illness, self-care abilities, interpersonal relationships, vocational functioning, and overall care needs [3]. FEP is commonly classified into three main categories: the first treatment contact, the duration of antipsychotic medication use, and the duration of psychosis [2].

Relapse is defined as a period of clinical deterioration lasting at least one week, often necessitating an increased dosage of antipsychotic medication, more frequent hospital admissions, or additional consultations [4]. While many FEP patients, such as those diagnosed with first-episode schizophrenia, initially respond well to treatment, relapse remains a common challenge and can significantly hinder long-term recovery [5]. Studies estimate that up to 80% of treated FEP patients experience relapse within the first five years [3], making relapse prevention a critical aspect of FEP management [5].

With each subsequent relapse, the risk of recurring psychotic symptoms increases, often leading to progressive grey matter loss and reduced effectiveness of antipsychotic treatments [6]. This deterioration results in worsening clinical outcomes for patients [3] and imposes significant financial and emotional burdens on patients, caregivers, and healthcare systems. Research indicates that the cost of managing relapse cases is approximately four times higher than that of non-relapse cases [7]. Additionally, relapses may prolong the duration of antipsychotic medication use [8].

Given these challenges, understanding the factors contributing to relapse is essential for improving FEP management. A systematic review and meta-analysis of longitudinal studies identified key predictors of relapse, including medication noncompliance, ongoing substance abuse, caregiver feedback, and poor premorbid adjustment. These factors increased the risk of relapse by 4-fold, 3-fold, 2.3-fold, and 2.2-fold, respectively [9].

Medication noncompliance is a significant global issue in schizophrenia treatment and is often one of the most challenging aspects of care [10]. A study by Al Maqbali et al. found that only 12% of schizophrenia patients completed a one-year treatment program [11]. The UK National Institute for Health and Care Excellence (NICE) guidelines recommend gradual antipsychotic withdrawal over at least two years, emphasizing close monitoring for relapse symptoms. Discontinuing antipsychotic treatment within the first two years of diagnosis is associated with a high risk of relapse [12].

Psychosocial factors also play a crucial role in relapse. A study by Suvisaari et al. found that a long duration of untreated psychosis (DUP), poor premorbid adjustment, and comorbid substance use disorders are linked to worse relapse outcomes [13]. Similarly, a four-year follow-up study by Qin et al. reported that patients with a prolonged DUP were more likely to experience frequent relapses requiring hospitalization, along with poorer social interactions, compared to those with a shorter DUP [14]. Moreover, Weibel et al. report that substance use is particularly common in FEP patients and is associated with a worse prognosis and higher relapse rates [15].

Family involvement is another critical factor in FEP treatment. Research indicates that family intervention programs significantly improve medication adherence, thereby reducing relapse risk [16,17]. Additionally, behavioral therapies that focus on enhancing stress-coping mechanisms have been shown to lower relapse rates in FEP patients [18].

By addressing these medication-related and psychosocial factors, targeted interventions can help mitigate the risk of relapse and improve long-term outcomes for individuals experiencing FEP.

Study aim and rationale

This study aimed to identify both medication- and non-medication-related factors contributing to relapse in FEP patients admitted to the Department of Behavioral Medicine (in either the inpatient or outpatient department) at Sultan Qaboos University Hospital (SQUH). Additionally, we sought to determine the key factors associated with noncompliance with antipsychotic medications.

Research on FEP relapse and its contributing factors remains limited in the Middle East. To address this gap, our study explored potential reasons for relapse among FEP patients at SQUH. By enhancing our understanding of these medication- and non-medication-related factors, we hope to inform future strategies for mitigating and potentially preventing relapse in FEP patients.

## Materials and methods

This retrospective cohort study examined FEP patients admitted to the inpatient and outpatient clinics of the Department of Behavioral Medicine at SQUH in Muscat, Oman. Data were extracted from patients’ medical records in Trakcare^®^, the hospital’s information system. Each patient’s medical history was thoroughly reviewed, including their date of arrival, age, gender, condition, relapse dates, relapse frequency, and overall past medical history (including substance misuse and mood symptoms). The study covered records from June 2006 to December 2019.

Inclusion and exclusion criteria

Patients were included if they had sufficient medical record information, experienced psychosis for the first time, and met the Diagnostic and Statistical Manual of Mental Disorders (DSM-V) criteria for a psychotic disorder. To ensure a true FEP diagnosis, patients must have presented to the psychiatry department for the first time and had no prior history of taking psychiatric medications. Both male and female patients were eligible.

Exclusion criteria included patients with a history of psychosis treated at another healthcare facility, those whose condition did not improve or relapsed before their first visit to SQUH, and individuals with psychosis due to organic medical causes (e.g., liver failure with encephalopathy).

Sociodemographic factors and possible risk factors associated with relapse

Patient records were analyzed to classify variables into demographic, medical, and treatment history categories. Demographic variables included age, gender, education level (primary, secondary, or tertiary), employment status (employed or unemployed), and place of residence, categorized as urban (Muscat and surrounding areas) or rural (other provinces). Medical and treatment history factors included past medical history, family history of mental disorders, use of traditional medicine, presence of substance abuse, hospitalization status (inpatient or outpatient), and whether the patient left against medical advice (LAMA). Follow-up patterns were categorized as regular (less than three months) or irregular (more than three months). Information was also gathered on the type of prescribed antipsychotic medication (typical or atypical), medication compliance, and reasons for noncompliance, which included symptom improvement, adverse drug reactions, lack of support, poor insight, and stigma. Admission at relapse was also documented.

The relapse rate was calculated based on the time between a return to baseline and symptom reemergence. Patients were classified into two groups based on whether relapse occurred within one year or beyond one year. Major life events were recorded according to the Holmes and Rahe Stress Scale, which includes significant stressors such as the death of a family member, divorce, major illness, marriage, job loss, pregnancy, and substantial changes in work.

Statistical analysis

Data were reviewed, coded, and analyzed using IBM SPSS Statistics for Windows, Version 23.0 (Released 2015; IBM Corp., Armonk, NY, USA). Descriptive statistics were used to determine frequencies and percentages for demographic and clinical variables. Each variable was categorized into two groups, and a chi-squared test was conducted to assess significant associations. The chi-squared test was selected because it is well suited for analyzing categorical data, such as relapse versus no relapse and medication compliance versus noncompliance. A p-value of less than 0.05 was considered statistically significant.

Ethical approval

Ethical approval was obtained from the Medical Research Ethics Committee at the College of Medicine and Health Sciences, SQUH, in September 2021 (MERC #2581).

## Results

A total of 213 FEP patients met the inclusion criteria. The study sample consisted of 122 female patients (57.3%) and 91 male patients (42.7%) who presented with nonorganic FEP. The diagnoses included brief psychotic disorder (58.2%), delusional disorder (10.8%), schizophrenia (9.0%), substance-induced schizophrenia (3.3%), schizoaffective disorder (5.6%), and bipolar disorder (13.1%).

The patients’ ages ranged from 12 to 55 years, with the majority falling within the 15- to 20-year age group (mean age = 25 years). Regarding marital status, 140 patients (65.7%) were single, while 73 (32.5%) were married.

The mean relapse rate across the sample was 14.6 months, with a minimum of two weeks and a maximum of five years. The associations between relapse rate and various sociodemographic and clinical variables are summarized in Table 1.

**Table 1:** Sociodemographic and clinical characteristics of FEP patients and their relapse rates FEP, first-episode psychosis; LAMA, left against medical advice

Variable	Categories	n (%)	Mean relapse rate in months	p-Value
LAMA	Yes	47 (22.0%)	9.95	0.001
	No	166 (78.0%)	15.95	
Residence	Urban	104 (48.4%)	17.05	0.005
	Rural	109 (51.6%)	12.31	
Employment status	Employed	66 (31.0%)	16.21	0.807
	Unemployed	147 (69.0%)	13.93	
Follow-up pattern	Regular	137 (64.3%)	14.35	0.601
	Irregular	76 (35.7%)	15.13	
Traditional healers	Yes	44 (20.7%)	13.84	0.508
	No	169 (79.3%)	14.83	
Substance misuse	Yes	15 (7.0%)	8.23	0.429
	No	198 (93.0%)	15.11	
Hospitalization status	Inpatient	138 (64.8%)	15.10	1.000
	Outpatient	75 (35.2%)	13.76	
Major life event	Present	139 (65.3%)	11.56	0.404
	Absent	74 (34.7%)	16.26	

Patients who had signed LAMA forms (n = 47, 22.0%) experienced a significantly shorter average relapse rate of 10 months compared to those who did not sign LAMA forms.

Patients residing in urban areas (n = 104, 48.8%) had a significantly longer mean relapse rate of 17.1 months compared to those in rural areas, who had an average relapse rate of 12.3 months (p = 0.005).

Among the study sample, the majority of patients were unemployed (n = 147, 69.0%), with an average relapse rate of 13.93 months, whereas employed patients had a slightly longer relapse rate of 16.21 months. However, this difference was not statistically significant (p = 0.807).

Regarding marital status, most patients were single (n = 140, 65.7%), with an average relapse rate of 16.21 months, compared to married patients, who had a relapse rate of 11.6 months. This difference was not statistically significant (p = 0.576, p > 0.05).

The majority of patients maintained a regular follow-up pattern (n = 137, 64.3%) with an average relapse rate of 14.4 months, compared to those with an irregular follow-up pattern (15.1 months). However, this difference was not significant (p = 0.601).

Patients who sought treatment from traditional healers before psychiatric care (n = 44, 20.7%) had an average relapse rate of 13.8 months, while those who did not (n = 169, 79.3%) had a slightly longer relapse rate of 14.8 months. This difference was not statistically significant (p = 0.508).

Patients with a history of illicit drug use (n = 15, 7.0%) had a shorter average relapse rate of eight months compared to those without substance use, who had an average relapse rate of 15 months. However, this relationship was not statistically significant (p = 0.429).

Regarding hospitalization status, most patients were hospitalized (n = 138, 64.8%), with no significant difference in relapse rates between inpatients and outpatients (p = 1.00).

**Figure 1:**
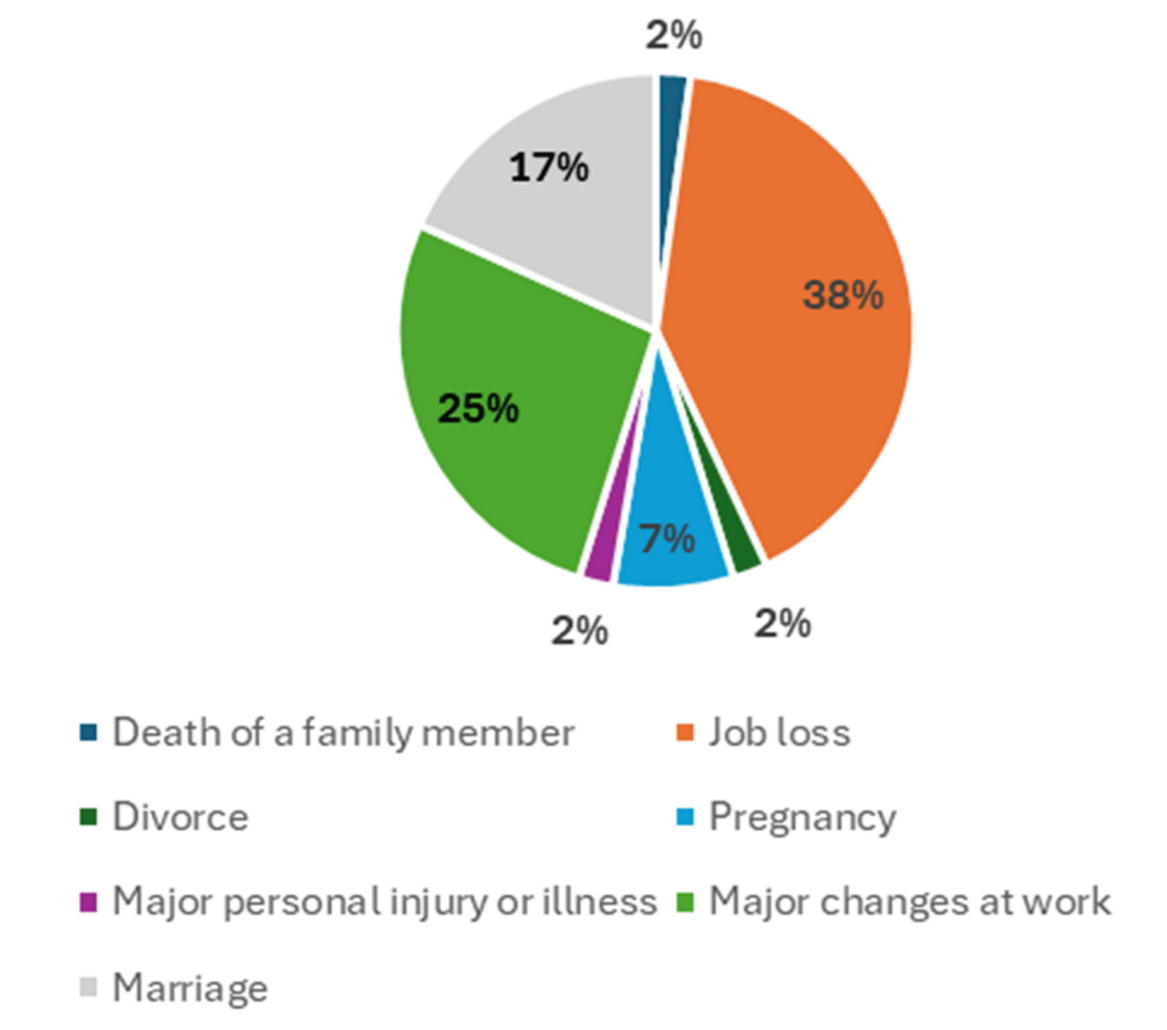
Percentage of patients who experienced a major life event based on the Holmes and Rahe Stress Scale

Additionally, patients who had experienced a major life event (n = 139, 65.3%) had a shorter average relapse rate of 11.6 months compared to those who had not (n = 74, 34.7%), who had an average relapse rate of 16.3 months. The most commonly reported major life event was job loss, as illustrated in Figure 1.

Nevertheless, the relationship between major life events and relapse rate was not statistically significant (p = 0.404). A total of 107 patients (50.23%) reported being noncompliant with their medication. Among these patients, the mean relapse time was 12 months (SD = 13.23), ranging from less than one month to a maximum of 85 months, with a median duration of nine months. In contrast, 106 patients (49.77%) were compliant with their medications, with a mean relapse time of 17.22 months (SD = 19.644), ranging from one month to a maximum of 108 months, and a median duration of 12 months. To further analyze the association between relapse and medication compliance, relapse time was categorized into two groups: “less than one year” and “more than one year.” The relationship between drug compliance and relapse was statistically significant (p = 0.03). The associations between drug noncompliance and demographic factors are summarized in Table 2.

**Table 2:** Demographic and clinical characteristics of FEP patients (N = 213) in relation to drug noncompliance p-Values were obtained using chi-squared tests. FEP, first-episode psychosis

Variable	N (%)	Noncompliance, n (%)	p-Value
Relapse time			
Less than one year	114 (53.52%)	66 (57.90%)	0.024
More than one year	99 (46.48%)	41 (41.4%)	
Gender			
Male	91 (42.5%)	49 (53.8%)	0.440
Female	122 (57.0%)	58 (47.5%)	
Age			
Less than 18	30 (14.1%)	12 (40%)	0.311
More than 18	183 (85.9%)	95 (51.9%)	
Marital status			
Single	140 (65.7%)	73 (52.14%)	0.34
Married	71 (33.3%)	34 (47.89%)	
Employment status			
Employed	66 (31%)	33 (50%)	1.00
Unemployed	147 (69.0%)	74 (50.34%)	
Residence			
Urban	114 (53.5%)	55 (48.23%)	0.627
Rural	99 (46.5%)	99 (46.5%)	
History of traditional healers			
Present	44 (20.7%)	22 (50%)	1.00
None	169 (79.3%)	85 (50.3%)	
History of substance abuse			
Present	15 (7%)	10 (66.6%)	0.293
None	198 (92.5%)	97 (49.0%)	
Types of psychotic medication			
Typical	17 (8%)	10 (58.8%)	0.627
Atypical	196 (92%)	97 (49.5%)	
Follow-up at the clinic			
Regular	137 (64.0%)	56 (40.9%)	0.000424
Irregular	76 (35.5%)	51 (67.1%)	
Admission at relapse			
Admitted	60 (28.8%)	34 (56.7%)	0.189
Not admitted	148 (71.2%)	69 (46.6%)	

It was found that there was no statistically significant association between age group and drug compliance (p = 0.812). In terms of gender distribution, more than half of the male patients were noncompliant with their medications (n = 49, 53.8%), whereas the proportion was lower among female patients (n = 58, 47.54%). However, this difference was not statistically significant (p = 0.44).

Among employed patients, half were noncompliant with their medications. Similarly, nonemployed patients exhibited nearly the same level of noncompliance (n = 147, 69%), with no statistically significant association between employment status and medication adherence.

Approximately half of the patients adhered to their medications, regardless of whether they had sought treatment from traditional healers (n = 22, 50%). As a result, no significant relationship was found between the history of consulting traditional healers and drug compliance (p = 1.00).

With respect to substance abuse history, 10 patients (66.67%) with a history of substance abuse were noncompliant, compared to five patients (33.3%) who had no history of substance abuse. However, this association was not statistically significant (p = 0.293).

Antipsychotic medications were classified as either typical or atypical. Atypical antipsychotics were prescribed to the majority of patients (n = 196, 92%) due to their lower likelihood of causing extrapyramidal side effects, whereas typical antipsychotics were prescribed to a smaller subset of patients (n = 17, 8%). The type of antipsychotic prescribed was not significantly associated with drug compliance (p = 0.56).

A significant association was observed between regular follow-up patterns and medication adherence (p = 0.0005). Most patients attended regular follow-up appointments at the clinic (n = 137, 64.0%). Among those with a regular follow-up pattern, 56 (40.88%) were noncompliant with their medication, compared to 51 (67.10%) who had irregular follow-up patterns (n = 76, 35.51%).

**Figure 2:**
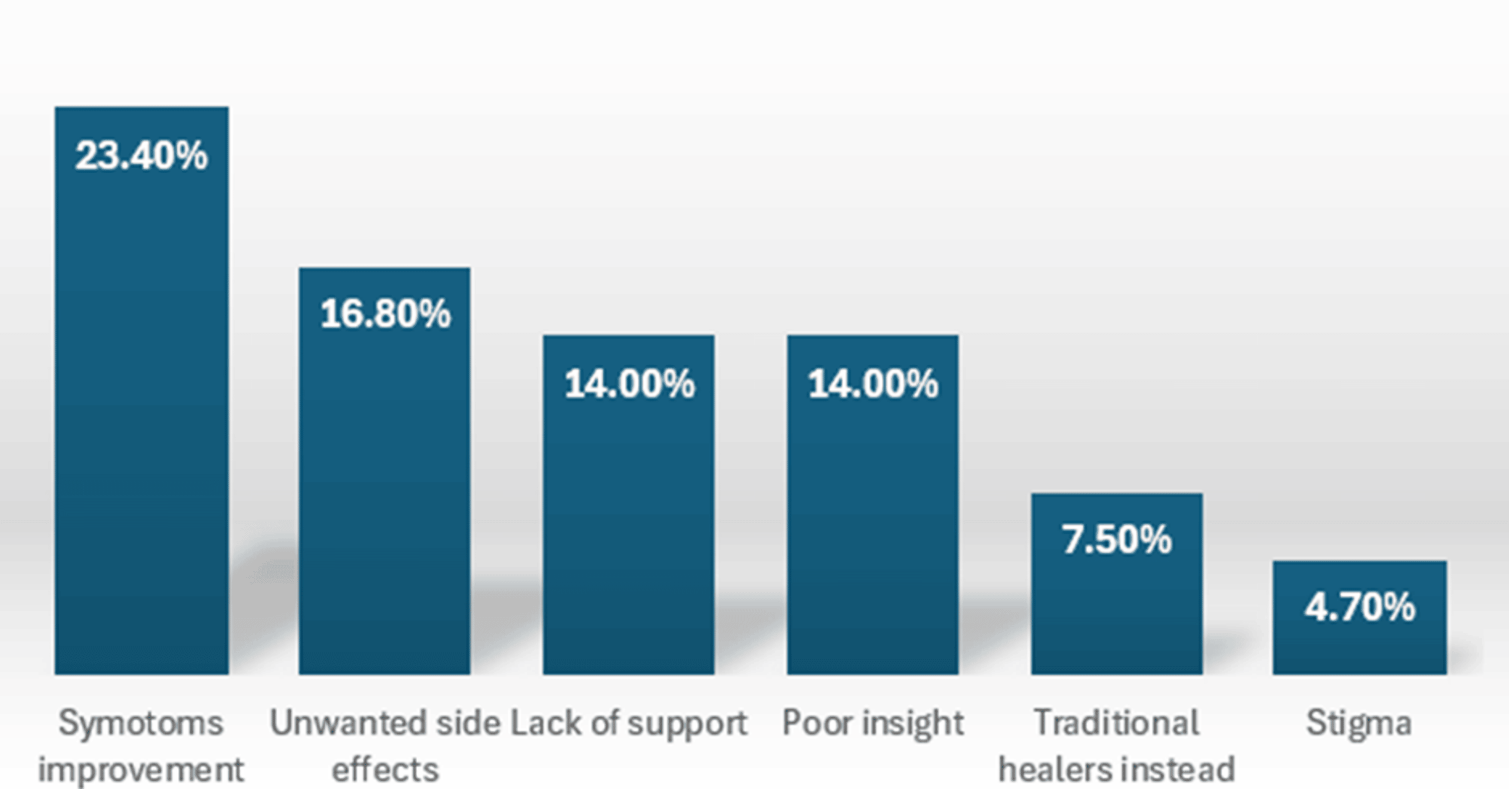
Reasons for medication noncompliance among patients with FEP FEP, first-episode psychosis

Regarding the reasons for noncompliance, 25 patients (23.36%) discontinued their medication due to perceived symptom improvement. Adverse drug reactions were cited as a reason for noncompliance by 18 patients (16.82%). Additionally, lack of support (n = 15, 14.02%), poor insight (n = 15, 14.02%), and stigma (n = 5, 4.67%) also played significant roles in medication nonadherence, as shown in Figure 2.

As a result of medication noncompliance, 34 (33.01%) noncompliant patients required hospital admission, compared to 26 (24.76%) compliant patients. However, this difference was not statistically significant (p = 0.246).

## Discussion

Non-medication-related reasons for relapse in patients with FEP

This study examined the sociodemographic and clinical profiles of Omani patients with FEP aged 12-55 years and their relationship to relapse rates. The patients were treated at the Behavioral Medicine Department of SQUH. Among the 213 study participants, the mean relapse rate was 14.6 months. The most significant factors associated with relapse rates were LAMA and place of residence.

Several factors may contribute to difficulties in adhering to treatment plans, leading to shorter relapse periods among Omani patients. Psychiatric services in Oman are not equally distributed across all provinces. Additionally, social stigma and cultural beliefs often act as barriers to continued psychiatric care [19]. These factors were reconsidered when analyzing the relationship between relapse rates and sociodemographic and clinical profiles. A significant association was found between a patient’s place of residence - urban versus rural - and relapse rates.

The study identified two key variables influencing relapse rates in FEP patients. First, a significant relationship was observed between a patient’s residence and relapse rate. Specifically, patients living in rural areas had significantly shorter relapse periods compared to those residing in urban areas. Notably, more than half of the study participants (n = 109, 51.6%) lived in rural regions of Oman.

Despite Oman’s advancements in public health over recent decades, the availability of mental health providers remains insufficient. The primary psychiatric hospitals, SQUH and Al-Massarah, are both located in urban areas [19,20]. Consequently, patients in rural areas face greater challenges in refilling their medications and attending follow-up appointments compared to urban residents. Expanding psychiatric services beyond Muscat, establishing mobile mental health clinics, and developing community outreach programs could help bridge this gap. Additionally, government-subsidized transportation for follow-up visits may improve patient compliance and long-term outcomes.

The study also found a significant relationship between relapse rates and LAMA. The most common reason for LAMA was dissatisfaction with treatment regimens, leading patients to seek care from traditional healers instead. This group demonstrated lower medication adherence and irregular follow-up visits, putting them at a higher risk of symptom relapse within a shorter time frame. The preference for traditional healing over psychiatric care may be linked to stigma surrounding mental illness in traditional societies, which can result in a lack of trust in biomedical psychiatric treatment [21]. Collaborating with traditional healers and religious leaders to develop culturally sensitive mental health awareness programs could encourage early psychiatric intervention. Additionally, training healthcare providers to address stigma-related concerns in a nonjudgmental manner may encourage patients to remain engaged in treatment.

Employment status was not significantly associated with relapse rates. This finding aligns with the results of Rinaldi et al., who reported that employment can increase stress levels in patients [22]. Moreover, medication compliance often results in sedative effects, making it difficult for patients to maintain employment. This, in turn, can lead to job loss and financial difficulties, both of which may increase the risk of relapse.

Seeking treatment from traditional healers is a common initial response when psychotic symptoms first appear. Previous studies have shown that relying on traditional healing methods is associated with a longer DUP [21]. However, in this study, there was no significant difference in relapse rates between patients who initially sought traditional healing and those who sought psychiatric care. One possible explanation is that many patients only turn to psychiatric treatment after traditional methods fail to improve their symptoms. As a result, these patients may become more committed to psychiatric care, potentially influencing relapse rates.

Although prior research has established a strong association between substance use and frequent psychotic relapses [15,18], this study did not find a statistically significant relationship between substance use and relapse. The majority of patients with a history of substance abuse - primarily marijuana users, with a small number of methamphetamine users - ceased substance use following treatment, which may explain the lack of a significant impact on relapse rates. Furthermore, none of the patients in this study developed new substance use disorders after their initial diagnosis, which may have also contributed to the absence of a significant association.

Fusar-Poli et al. emphasized the importance of an integrative treatment approach beyond antipsychotic medications to reduce relapse risk in patients with substance use disorders. This includes cognitive behavioral therapy (CBT) and family psychoeducation, both of which were implemented in this study [18]. Psychoeducation sessions involved structured meetings with patients and their families to enhance medication adherence, develop coping strategies for symptom management, and improve understanding of psychosis. These sessions also aimed to strengthen family support and reduce stigma, both of which have been associated with better long-term outcomes. Additionally, CBT was employed to help patients identify and modify maladaptive thought patterns related to substance use and psychosis. Patients were trained in stress-reduction techniques, relapse prevention strategies, and craving management. The effectiveness of this multidisciplinary approach was reflected in improved medication adherence, reduced substance use, and lower relapse rates.

The study found no significant association between hospitalization status and relapse rates. This finding is consistent with Addington et al., who suggested that the relationship between hospitalization and relapse rates is characterized by high sensitivity but low specificity [23]. While major life events have been proposed as potential triggers for relapse and increased severity of psychotic symptoms [23], this study did not confirm a significant relationship between relapse rates and life stressors, similar to findings from previous research [24].

Some studies argue that life events do not directly influence individuals’ perceived stress levels in psychosis. Instead, such events may heighten emotional reactivity to daily stressors, which, in turn, could impact remission depending on the individual’s history of life events and vulnerability [24]. Research has also shown that life stressors are less likely to trigger relapse in patients who are no longer on antipsychotic medications, particularly in low-expressed emotion environments, unless a major life event occurs [25]. Additionally, the recurrence of psychotic symptoms is more strongly influenced by continued antipsychotic treatment than by life stressors alone [25].

Medication-related reasons behind relapse in patients with FEP

Antipsychotic medications are essential for managing psychosis, making adherence crucial in preventing symptom recurrence and relapse. In this study, the average relapse rate among patients who were noncompliant with their medications was approximately 12 months, compared to 17.22 months for those who adhered to their prescribed treatment regimen.

Previous research has shown that noncompliance with antipsychotic medication significantly increases relapse risk. One study on patients with chronic schizophrenia reported that relapse rates due to medication nonadherence reached 50% within a year following hospital discharge [26]. Treatment outcomes heavily depend on medication adherence, which can be influenced by various factors [13]. However, our study found no significant associations between medication compliance and sociodemographic factors such as gender, age, family or marital status, and employment status. These findings align with a meta-analysis that similarly reported no significant relationship between these variables and medication adherence in most of the included studies [10].

The prevalence of noncompliance in our study (50.23%) was relatively high compared to reports from other studies, such as one conducted in London, which found a noncompliance rate of approximately 40% [26]. This difference may be attributed to limited awareness and poor insight regarding mental health disorders and treatment among the Omani population. A 2020 study in Oman that assessed medication adherence among 151 psychiatric patients at a tertiary care hospital in Muscat found that 31.1% of participants were not convinced they had a mental health condition, while 21.2% believed they did not require treatment. Additionally, 83.4% of participants did not know the name of their prescribed medications, and 42.4% reported that they had not received adequate information about their treatment [11].

Several factors contributed to medication noncompliance in our study. Among noncompliant patients, 23.36% stopped taking their medication after experiencing symptom improvement, despite research emphasizing the necessity of remaining on medication for at least two years after diagnosis, even if symptoms subside. Additionally, 16.82% cited adverse drug reactions - especially sedation - as a barrier to adherence. These findings are consistent with a prospective study that found that approximately 35% of patients considered adverse drug effects a major obstacle to medication adherence [27]. Another survey also identified persistent side effects, particularly weight gain in women and excessive sedation, as significant factors influencing medication noncompliance [28].

Lack of support was another key factor, affecting 14.02% of noncompliant patients. This was often seen in patients who ran out of medication or received unsupervised treatment, leading them to forget their doses. A prospective study similarly found that difficulty in maintaining a regular medication schedule was one of the most common barriers to adherence [29]. Additional factors influencing noncompliance included stigma (4.67%) and poor insight (14.02%), where some patients refused medication outright and were uncooperative with treatment instructions.

This study identified a significant association between regular follow-up appointments and medication adherence. Among the 76 patients (35.51%) who were lost to follow-up, 51 (67.10%) were noncompliant with their medication. These findings are consistent with a 2020 Omani study that assessed medication adherence among psychiatric patients at Al-Masarra Hospital, a major tertiary care center [11]. Regular follow-up visits, typically scheduled every three months or as needed, are critical in ensuring continued medication adherence. However, one of the primary reasons for missed follow-up appointments is the long travel distances to Oman’s two main psychiatric hospitals, SQUH and Al-Masarra, both located in Muscat. Many patients must travel extensive distances to access care, often requiring them to miss work, school, or college, which can further contribute to poor follow-up rates [20].

In this study, 33.01% of noncompliant patients required hospital admission due to relapse, compared to 24.76% of compliant patients. However, the association between medication adherence and hospitalization was not statistically significant. This contrasts with findings from most studies, which consistently show a strong link between lower adherence rates and increased hospitalization risk. For example, a retrospective study found that patients who maintained over 70% adherence to their prescribed medication regimen had significantly lower hospitalization rates compared to nonadherent patients [29].

Study limitations

This study had several limitations. First, as a retrospective study, the completeness of patient records varied, which may have influenced the results. Additionally, medication compliance was assessed based on information provided by relatives, with no objective method to verify its accuracy. This reliance on subjective reports introduces the possibility of bias or misinformation. Lastly, since the study was conducted at a single hospital, the findings may not be fully generalizable to other settings.

## Conclusions

Psychosis is a key symptom of various neurological and psychiatric conditions, significantly affecting a patient’s quality of life. This study examined the sociodemographic and clinical factors associated with relapse rates among FEP patients in Oman. Our findings highlight a strong correlation between relapse rates and both LAMA and place of residence (rural vs. urban). Additionally, patients who regularly attended follow-up appointments demonstrated better medication compliance than those who did not, emphasizing the critical role of continued engagement with mental health services.

To improve patient outcomes, mental health professionals in Oman should prioritize expanding access to psychiatric care in rural areas. This could include establishing more accessible mental health facilities and enhancing transportation options to encourage adherence to follow-up appointments. Additionally, integrating community-based care models - such as family-involved psychoeducation programs - can help reduce stigma and provide stronger support for FEP patients. Strengthening early intervention strategies, including outreach initiatives for at-risk individuals, may further reduce relapse rates. Future research should expand the sample size by including patients from Al-Massarah Hospital in Oman and conducting prospective cohort studies to track patients over time and identify modifiable relapse risk factors. Further investigations should explore the impact of socioeconomic determinants, substance use, and co-occurring mental disorders, as well as the effectiveness of early intervention programs, psychosocial therapies, and medication adherence. These insights could inform evidence-based policy changes aimed at enhancing long-term care and support for FEP patients in Oman.
